# Berberine Slows the Progression of Prediabetes to Diabetes in Zucker Diabetic Fatty Rats by Enhancing Intestinal Secretion of Glucagon-Like Peptide-2 and Improving the Gut Microbiota

**DOI:** 10.3389/fendo.2021.609134

**Published:** 2021-05-07

**Authors:** Ying Wang, Haiyi Liu, Miaoyan Zheng, Yanhui Yang, Huizhu Ren, Yan Kong, Shanshan Wang, Jingyu Wang, Yingying Jiang, Juhong Yang, Chunyan Shan

**Affiliations:** ^1^ National Health Council (NHC) Key Laboratory of Hormones and Development, Tianjin Key Laboratory of Metabolic Diseases, Chu Hsien-I Memorial Hospital and Tianjin Institute of Endocrinology, Tianjin Medical University, Tianjin, China; ^2^ Department of Pediatrics, Cangzhou People’s Hospital, Cangzhou, China

**Keywords:** berberine, intestinal microbiota, type 2 diabetes mellitus, glucagon-like peptide-2, intestinal permeability

## Abstract

**Background:**

Berberine is a plant alkaloid that has multiple beneficial effects against intestine inflammation. In our previous study, we have found that berberine also possesses an antidiabetic effect. However, whether berberine is useful in the prevention of type 2 diabetes mellitus (T2DM) through its effect on intestine endocrine function and gut microbiota is unclear.

**Aim:**

To investigate the effects of berberine in the prevention of T2DM, as well as its effects on intestine GLP-2 secretion and gut microbiota in ZDF rats.

**Methods:**

Twenty Zucker Diabetic Fatty (ZDF) rats were fed a high-energy diet until they exhibited impaired glucose tolerance (IGT). The rats were then divided into two groups to receive berberine (100 mg/kg/d; berberine group) or vehicle (IGT group) by gavage for 3 weeks. Five Zucker Lean (ZL) rats were used as controls. Fasting blood glucose (FBG) was measured, an oral glucose tolerance test was performed, and the Homeostatic Model Assessment of Insulin Resistance (HOMA-IR) was calculated. Intestinal expression of TLR-4, NF-κB, TNF-α, mucin, zona occludens-1 (ZO-1) and occludin were assessed (immunohistochemistry). Plasma levels and glutamine-induced intestinal secretion of glucagon-like peptide-1 (GLP-1) and GLP-2 were measured (enzyme-linked immunosorbent assay). The plasma lipopolysaccharide (LPS) level was measured. Fecal DNA extraction, pyrosequencing, and bioinformatics analysis were performed.

**Results:**

After 3 weeks of intervention, diabetes developed in all rats in the IGT group, but only 30% of rats in the berberine group. Treatment with berberine was associated with reductions in food intake, FBG level, insulin resistance, and plasma LPS level, as well as increases in fasting plasma GLP-2 level and glutamine-induced intestinal GLP-2 secretion. Berberine could increase the goblet cell number and villi length, and also reverse the suppressed expressions of mucin, occludin, ZO-1 and the upregulated expressions of TLR-4, NF-κB and TNF-α induced in IGT rats (P<0.05). Berberine also improved the structure of the gut microbiota and restored species diversity.

**Conclusion:**

Berberine may slow the progression of prediabetes to T2DM in ZDF rats by improving GLP-2 secretion, intestinal permeability, and the structure of the gut microbiota.

## Introduction

Type 2 diabetes mellitus (T2DM) is a metabolic disease characterized by hyperglycemia and insulin resistance (1). The increasing prevalence of T2DM, particularly in Asia, due to rapid industrialization/urbanization and associated lifestyle changes, is recognized as a major health problem (2–4). It has been estimated that around 382 million people worldwide were living with DM in 2013 and that this will rise to nearly 600 million by 2035 (5). In addition to those with T2DM, many people are diagnosed with prediabetes, and there were an estimated 374 million people with impaired glucose tolerance (IGT) in 2017 (6). China is the country with the highest total number of people with T2DM (5), with one survey estimating that China had around 114 million adults with T2DM and an additional 493 million with prediabetes in 2010 (7). Prediabetes is a high-risk state for the future development of diabetes: around 10% of patients with prediabetes develop T2DM annually, and around 70% will eventually develop T2DM during their lifetime (8).

Recent work has highlighted an important role of the incretin axis and gut microbiota in the pathophysiology of prediabetes (9). The microbiota is now recognized as a functional ‘organ’, and its composition and function are thought to contribute to host glycemic regulation and insulin sensitivity (10, 11). The incretin axis is one of the key biological molecular mechanisms underlying the impact of the gut microbiota on host glycemic control (12). Glucagon-like peptide-1 (GLP-1) is a gut-derived incretin hormone that stimulates insulin secretion, suppresses glucagon secretion, inhibits gastric emptying, and reduces appetite and food intake (11). Notably, patients with prediabetes have decreased plasma levels of GLP-1 (13, 14). Furthermore, the antidiabetic drug liraglutide, which acts as a GLP-1 receptor agonist, has been shown to significantly lower body weight and improve glucose metabolism while modifying the composition of the gut microbiota (15). In addition, incretin-based therapies may slow the progression or delay the onset of T2DM in patients with prediabetes, possibly by preserving β-cell function and mass and thereby helping to maintain good long-term metabolic control (16). GLP-2 is another gut-derived hormone and is secreted in response to nutrients and mucosal injury (17). GLP-2 reduces enterocyte apoptosis, enhances crypt cell proliferation in the small intestine, promotes intestinal cell proliferation, and confers resistance to cellular injury, thereby helping to maintain mucosal integrity, mucosal morphology, and intestinal barrier function (18, 19). However, recent experimental data suggest that GLP-2 also exerts beneficial effects on glucose metabolism (17, 20–22).

Berberine, an isoquinoline alkaloid originally isolated from the Chinese herb *Coptis chinensis*, has been used to treat diarrhea for many years in China (23, 24). Recently, researchers have found that berberine improves insulin resistance and endotoxemia in rat models of diabetes and patients with T2DM (25–28), and these actions may be related to the effects of berberine on the gut microbiota or gut mucosal integrity (29, 30). In our previous study, we found that berberine protects the intestinal barrier and mucosal integrity and helps to maintain GLP-2 secretion by intestinal L cells (31). Therefore, we hypothesized that berberine might protect against the development of T2DM by maintaining GLP-2 secretion, intestinal permeability, and the structure of the gut microbiota. The aim of this study was to investigate the effects of berberine on GLP-2 secretion, intestinal permeability, and the gut microbiota in a rat model of T2DM.

## Materials and Methods

### Rat Model of T2DM

A total of 20 male Zucker diabetic fatty (ZDF; fa/fa) rats and 5 male Zucker lean (ZL; fa/+) rats aged 6 weeks (190–210 g) were purchased from Beijing Vital River Laboratory Animal Technology Co. Ltd. [Beijing, China; animal license no: SCXK (Beijing) 2012-0001]. The animals were housed and maintained under standard laboratory conditions (12:12 h light-dark cycle; 20 ± 2°C temperature; 40–60% humidity; noise < 50 dB) at the animal house of the Institute of Biomedical Engineering, Chinese Academy of Medical Sciences, Tianjin, China. All experiments in the current study followed the ethical principles approved by the Animal Ethics Committee of Tianjin Medical University (approval # DXBYY-IACUC-2020023). After acclimatization, rats were fed a high-energy rodent diet (Purina 5008 rat chow; Charles River Laboratories, Wilmington, MA, USA) and water ad libitum. An oral glucose tolerance test (OGTT) was performed weekly to determine the onset and stage of experimental T2DM in the rats so that fasting blood samples from the medial canthus and fecal samples could be taken at the stages of normal glucose tolerance (NGT), IGT and T2DM.

### Grouping

The ZDF rats were randomly divided (using a random number table) into an IGT group (n = 10) and a berberine group (n = 10). The ZL rats were defined as the control group (n = 5). Rats in the berberine group were administered berberine hydrochloride (Jinke Pharmaceutical Company, Yunnan, China) by gavage at a dose of 100 mg/kg/d for 3 weeks (32), whereas rats in the IGT and control groups were given the same volume of vehicle (distilled water).

### Biochemistry Markers and OGTT

Rats were fasted for 12 hours (from 20:00 to 8:00 the next day) once weekly so that biochemistry investigations and an OGTT could be performed. First, blood was drawn from the tail vein (after disinfection of the tail with 70% isopropyl alcohol) using sterile blood collection tubes for the measurement of fasting blood glucose (FBG; One Touch Ultra blood glucose meter; Johnson & Johnson, New Brunswick, NJ, USA) and other biochemical parameters (see below). Then, an OGTT was performed to stage the development of T2DM and identify when all rats in the IGT group had progressed to T2DM. The rat was administered 50% glucose (2 g/kg), and the glucose levels in tail vein blood were measured at 30 min, 60 min, and 120 min. The following diagnostic criteria were used to define IGT (one criterion met) and T2DM (both criteria met): a blood glucose level at 2 hours after glucose loading > 11.1 mmol/L; and peak blood glucose level > 16.7 mmol/L (33). Glucose tolerance was evaluated by calculation of the area under the curve (AUC) using the trapezoidal area method: AUC = ¼ × FBG value + ½ × 0.5-hour glucose value + ¾ × 1-hour glucose value + ½ × 2-hour glucose value.

The fasting plasma levels of GLP-1, GLP-2, lipopolysaccharide (LPS), insulin, and glucagon were assessed using enzyme-linked immunosorbent assay (ELISA) kits (Hermes Criterion Biotechnology, Vancouver, BC, Canada) and a monochromator-based multi-mode microplate reader (SynergyMx; BioTek, Winooski, VT, USA) according to the manufacturer’s protocol. The insulin resistance index (Homeostatic Model Assessment of Insulin Resistance, HOMA-IR) was calculated as: HOMA-IR = (FBG level × fasting plasma insulin level)/22.5. The homeostatic model assessment of insulin secretion (HOMA-β) was calculated as HOMA-β = 20×fasting plasma insulin level/(FBG level-3.5).

An automatic biochemical analyzer (Modular DP800; Roche, Basel, Switzerland) was used for the determination of blood urea nitrogen (BUN) level and the plasma levels of alanine aminotransferase (ALT), aspartate aminotransferase (AST), creatinine (Cr), uric acid (UA), triglycerides (TG), total cholesterol (CHO), and high-density lipoprotein (HDL). Low-density lipoprotein (LDL) levels were determined using the Friedewald equation: LDL=CHO-HDL-TG/5.

### Resection of the Rat Small Intestine for Immunohistochemistry and Cell Culture Experiments

After the last OGTT had been performed, the rats were fasted for 24 hours and then sacrificed. Segments of the distal ileum (1–2 cm above the junction with the cecum) were removed and flushed in ice-cold phosphate-buffered saline (PBS). Some of the small intestinal segments were fixed with 4% formaldehyde (in buffer) for use in immunohistochemistry experiments, some of the small intestinal segments of distal ileal were embedded and frozen in OCT compound, then snap-frozen in liquid nitrogen, and stored at −80°C, and the other segments of ileal tissue were used for cell culture.

### Histological Analyses of Rat Small Intestinal Tissue

The distal ileum tissues from the rats were fixed with formalin, embedded in paraffin, and sectioned into 4-μm-thick sections. Hematoxylin and eosin (H&E) staining was performed following standard methods. The slides were observed under a microscope with a digital camera. The morphometric analysis of villi length and goblet cells was undertaken using Image-Pro Plus (Media Cybernetics, Inc., Rockville, MD, USA) by a blinded investigator.

### Evaluation of the Expression of Zona Occludens-1 (ZO-1) and Occludin in Intestinal Tissue

Segments of distal ileum were fixed with formalin, embedded in paraffin, sectioned into 4-μm-thick slices, and subjected to immunohistochemistry (IHC) to detect ZO-1 and occludin. The sections were deparaffinized, rehydrated, and incubated with hydrogen peroxide for 10 min at room temperature to block endogenous peroxidase activity. After incubation for 20 min in normal goat serum, sections were immunostained using goat anti-ZO-1 polyclonal primary antibody (ab190085; Abcam, Cambridge, UK) or rabbit anti-occludin polyclonal primary antibody (ab31721; Abcam, Cambridge, UK) together with a streptavidin-biotin-peroxidase detection system and a DAB reagent kit (Zhongshan Jinqiao Biotechnology Company, Beijing, China). The sections were then stained by hematoxylin, thoroughly washed, dehydrated, and mounted using coverslips and DPX mounting medium. The slides were observed under a microscope (#BX51T-PHD-J11, Olympus Corporation, Tokyo, Japan). The number of immunopositive cells in 5 fields of view was counted independently by two researchers who were blinded to the grouping. The number of positive cells (value A) was graded as 0 (0–1%), 1 (1–10%), 2 (10–50%), 3 (50–80%), or 4 (80–100%). The staining intensity (value B) was graded as 0 (negative), 1 (weakly positive), 2 (positive), or 3 (strongly positive). The IHC score was then calculated as IHC = A × B (34).

### Evaluation of the Expression of TLR-4, NF-κB, TNF-α and Mucin in Intestinal Tissue

Cryostat sections cut at 6–7 microns. After 4% paraformaldehyde fixation and antigen repair, the tissues were treated with 3% H_2_O_2_ for 10 min, blocked with 1% bovine serum albumin for 1 h, and then incubated with specific primary antibodies against TLR-4 (ab22048; Abcam, Cambridge, UK), NF-κB (ab16502; Abcam, Cambridge, UK), TNF-α (ab6671; Abcam, Cambridge, UK), and Mucin 2 (ab272692; Abcam, Cambridge, UK) at 4°C overnight. After washing, the sections were incubated with the secondary antibody for 1 h at 37°C. Finally, diaminobenzidine was added to the slides, which were then counterstained with hematoxylin. Pathological changes in tissue and protein expression were viewed under a light microscope with a digital camera. For western blots, proteins from tissues were extracted with RIPA buffer containing a protease inhibitor cocktail, and the protein concentration was determined by a BCA protein assay kit (Solarbio, China). Then, 40 μg of protein was separated by SDS/PAGE, transferred to nitrocellulose filter membranes, and blocked with 5% nonfat milk for 1 h at room temperature, and then the membranes were incubated with the following primary antibodies against mucin (ab272692; Abcam, Cambridge, UK) at 4°C overnight. After the blots were washed, the secondary antibody was added and incubated for 1 h at room temperature. The immunoblots were detected with an ECL kit (Advansta, United States). Band intensity was analyzed using ImageJ software and normalized to the expression of GAPDH.

### Evaluation of the Capacity of Intestinal L Cells to Secrete GLP-1 and GLP-2

Three-centimeter lengths of distal ileum were kept in PBS for the determination of glutamine-stimulated GLP-1 and GLP-2 secretion. The ileum was cleared of connective tissue, cut open, and rinsed with PBS and cell culture medium, and the outer muscle layers were carefully stripped off. Subsequently, the ileum was cut into three one-centimeter-long segments. Each segment was chopped using eye scissors and transferred to a 24-well plate, and 1000 mL of pre-warmed Roswell Park Memorial Institute (RPMI) 1640 cell culture medium (containing 2.05 mM glutamine) was added. The tissues were incubated for 60 min at 37°C in a humidified incubator at 5% CO_2_, and then 4 mM glutamine was added to each well to stimulate the secretion of GLP-2. The tissue pieces were removed after 30 min, and the conditioned medium was stored at -80°C until use. The concentrations of GLP-1 and GLP-2 in the medium were assayed using ELISA kits (Hermes Criterion Biotechnology) (31).

### Fecal DNA Extraction and Pyrosequencing

The rats were placed on a horizontal test bench, the tail was lifted, and the abdomen was gently massaged to stimulate defecation. A clean 4-ml centrifuge tube was placed near the anus to take the feces. The feces were placed in liquid nitrogen immediately after the tube was sealed, and then stored in the refrigerator at -80°C for fecal DNA extraction and analysis. The extraction of fecal DNA from stool samples (180–220 mg) was carried out using the TIANamp Stool DNA kit (Tiangen Biotech, Beijing, China). DNA yields were measured from the ratio of the absorbances at 260 nm and 280 nm (pure DNA has a 260/280 nm absorbance ratio of 1.7–1.9) using NanoDrop spectrophotometry (Maestrogen, Hsinchu City, Taiwan). The DNA extracted from each stool sample was used as a template for the amplification of the V4 region of 16S rDNA genes. A dual-index sequencing approach was used to allow the generation of a large number of high-quality sequences while minimizing the cost of long and customized primers. Polymerase chain reaction (PCR) was performed using the V4 Dual-index Fusion PCR Primer Cocktail (515F:5’-GTGCCAGCMGCCGCGGTAA-3’, 806R:5’-GGACTACHVGGGTWTCTAAT-3’ and Phusion High-Fidelity PCR Master Mix (New England Biolabs, Ipswich, MA, USA) with a melting temperature of 56°C and 30 cycles. The PCR products were purified with Agencourt AmpureXP beads (Beckman Coulter, Brea, CA, USA) to remove unspecific products. The final library was quantified by determination of the average molecule length (Agilent 2100 Bioanalyzer Instrument and Agilent DNA 1000 Reagents; Agilent, Santa Clara, CA, USA) and quantitative PCR (EvaGreen dye, Biotium, Hayward, CA, USA). Paired-end sequencing was performed on a MiSeq platform (Illumina, San Diego, CA, USA) with the PE250 (PE251+8+8+251) sequencing strategy (MiSeq Reagent Kit). All procedures for fecal microbe analysis were performed under sterile conditions.

### Bioinformatics Analysis

Raw pyrosequencing reads generated from the sequencer were quality filtered into Clean Data, and paired-end reads were assembled using FLASH (Fast Length Adjustment of SHort reads) v1.2.11. High-quality valid reads were clustered into operational taxonomic units (OTUs) with 97% similarity cutoff using USEARCH v7.0.1090. The phylogenetic affiliation of each OTU was analyzed by RDP Classifier v2.2 against the Greengene (V201305), Silva (V119), and UNITE (Version 6 20140910) databases using a confidence threshold of 80%. The representative sequences of the OTUs were used to analyze the alpha diversity (rarefaction curve analysis, observed species index, and Shannon diversity index) on the basis of their relative abundance (Mothur v1.31.2). Statistical comparisons among groups were made using the Kruskal-Wallis Test. A heatmap was generated according to the relative abundance of the OTUs using R v3.1.1. Weighted and unweighted UniFrac distance metric analysis was performed using OTUs for each sample, and principal coordinates analysis (PCoA) was conducted according to the matrix of distance.

### Statistical Analysis

Continuous measurement data are expressed as the mean ± standard error of the mean (S.E.M.) and compared between groups using one-way analysis of variance (ANOVA) and the Least Significance Difference (LSD) *post hoc* test. Count data are expressed as the median (four-point interval) and percentage and compared between groups using the rank-sum test. A P-value < 0.05 was taken to indicate a significant difference.

## Results

### Effects of Berberine on the Incidence of T2DM in ZDF Rats

All ZDF rats had NGT at 6 weeks of age but exhibited IGT at 9 weeks of age. After 3 weeks of the intervention (i.e., berberine or vehicle, depending on the group), all rats in the IGT group (100%) developed T2DM, whereas only 3 rats (30%) in the berberine group progressed to T2DM, with 3 rats (30%) remaining in the IGT stage and 4 rats (40%) recovering to NGT. No rats died during the study.

### Effects of Berberine on FBG Level, the OGTT and Plasma Levels of Insulin and Glucagon

**Figure 1:**
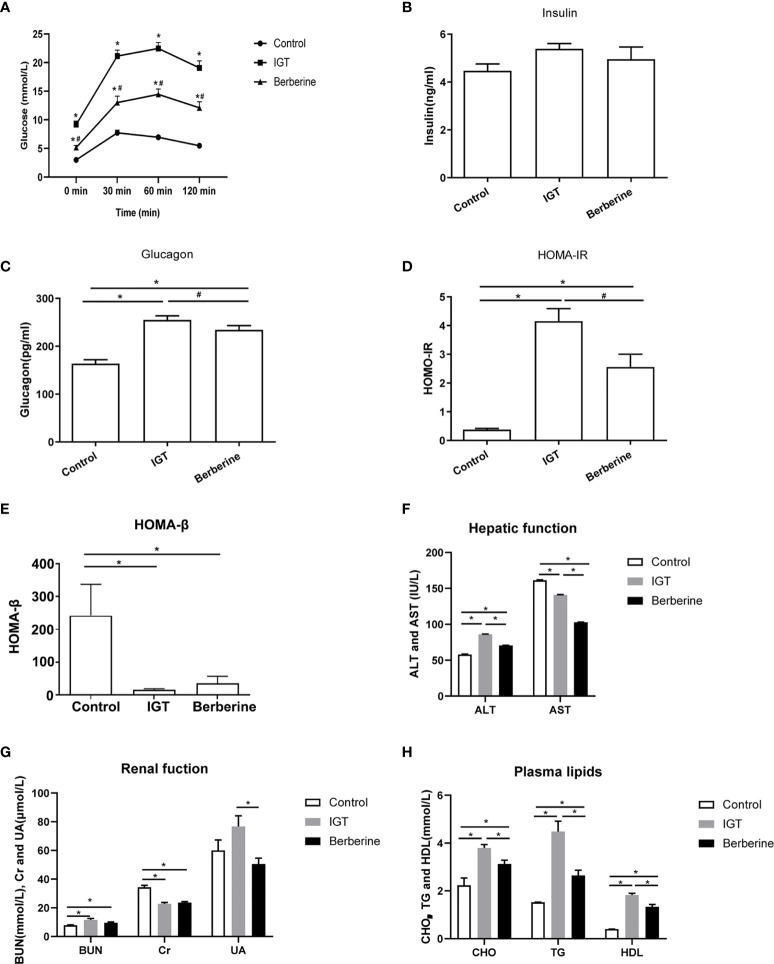
Effects of berberine on biochemistry parameters. **(A)** Oral glucose tolerance test after 3 weeks of intervention. *P < 0.05 *vs.* control group, ^#^P < 0.05 vs. IGT group. **(B)** Fasting plasma insulin level. **(C)** Fasting plasma glucagon level. *P < 0.05 vs. control group, ^#^P < 0.05 *vs.* IGT group. **(D)** Homeostatic Model Assessment of Insulin Resistance (HOMA-IR). *P < 0.05 *vs.* control group, ^#^P < 0.05 *vs.* IGT group. **(E)** Homeostatic model assessment of insulin secretion (HOMA-β). *P < 0.05. **(F)** Plasma levels of alanine aminotransferase (ALT) and aspartate aminotransferase (AST). *P < 0.05. **(G)** Blood urea nitrogen (BUN), plasma creatinine (Cr) and plasma uric acid (UA). *P < 0.05. **(H)** Plasma levels of cholesterol (CHO), triglycerides (TG) and high-density lipoprotein (HDL). *P < 0.05. Control group (n=5), IGT group (n=10), and berberine group (n=10).

FBG level was significantly higher in the IGT group than in the control group (9.38 ± 1.66 mmol/L *vs.* 3.52 ± 1.13 mmol/L, P < 0.05). The FBG level in the berberine group (5.03 ± 1.26 mmol/L) was significantly lower than that in the IGT group (P < 0.05) but still higher than that in the control group (P < 0.05). Oral glucose tolerance, measured by the AUC, was significantly worse in the IGT group than in the control group (39.82 ± 6.07 *vs.* 12.54 ± 0.86, P < 0.05; **Figure 1A**). Furthermore, oral glucose tolerance in the berberine group (24.68 ± 5.24) was significantly better than that in the IGT group (P < 0.05), although it remained significantly worse than that in the control group (P < 0.05; **Figure 1A**).

There were no differences between groups in the fasting plasma insulin level (**Figure 1B**). However, plasma glucagon level was significantly higher in the IGT group than in the control group (P < 0.05; **Figure 1C**). Treatment with berberine significantly reduced the plasma glucagon level (P < 0.05), although it remained at an elevated level in comparison to the control group (P < 0.05; **Figure 1C**). HOMA-IR was markedly increased in the IGT group as compared with the control group (P < 0.05; **Figure 1D**). Moreover, HOMA-IR was significantly decreased following treatment with berberine (P < 0.05), although it was higher than that in the control group (P < 0.05; **Figure 1D**). HOMA-β was markedly reduced in the IGT group compared with the control group(16.06 ± 2.94 vs 241.98 ± 95.07, P < 0.05), but there were no differences between the IGT and berberine groups (**Figure 1E**).

As can be observed in **Supplementary Figure 2**, the berberine group showed a decreased food intake by 2 weeks compared with the IGT group, but body weight was almost the same in the two groups. In addition, the berberine group had markedly improved blood glucose levels compared with the IGT group.

### Effects of Berberine on Hepatic Function, Renal Function, and Plasma Lipids

The IGT group had a higher ALT level and a lower AST level than the control group (P < 0.05), while treatment with berberine was associated with significant reductions in both ALT and AST (P < 0.05; **Figure 1F** and **Supplementary Table 1**). ZDF rats had a higher BUN level and a lower Cr level than ZL rats (P < 0.05), with no significant differences between the IGT and berberine groups (**Figure 1G** and **Supplementary Table 1**). The levels of CHO, TG, and HDL were higher in the IGT group than in the control group (P < 0.05), and treatment with berberine was associated with improvements in all three parameters (P < 0.05; **Figure 1H** and **Supplementary Table 1**), but there was no improvement in LDL.

### Effects of Berberine on the Intestinal Mucosal Barrier

**Figure 2:**
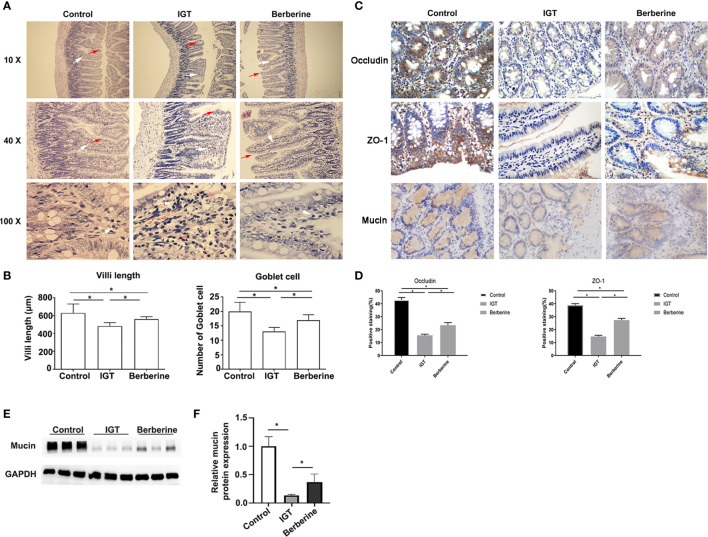
Effects of berberine on the intestinal mucosal barrier. **(A)** Representative hematoxylin-eosin staining. Inflammatory cell infiltration, broadening and fusion of villi were indicated using arrows. Magnification 10 ×, 40 ×, and 100 × respectively of each row from top to bottom in **(A)**. **(B)** Analysis of the villi length and the numbers of goblet cells. *P < 0.05. **(C)** Immunohistochemistry images of the intestinal epithelium and glandular cells showing the distribution of tight-junction proteins and mucin 3 weeks after the intervention. Magnification 400 × in **(C)**. **(D)** Statistical analysis of scored immunohistochemistry positive staining for occludin and ZO-1. *P < 0.05. **(E)** Effects of berberine on mucin by western blotting. **(F)** Statistical analysis of mucin expression by western blotting. *P < 0.05. Control group (n=5), IGT group (n=10), and berberine group (n=10).

The IGT group exhibited evidence of damage to the intestinal mucosa, including a disorderly arrangement of irregular villi, broadening and fusion of villi, reduced villi length, reduced numbers of goblet cells and inflammatory cell infiltration into the lamina propria (**Figures 2A, B**). However, treatment with berberine improved the intestinal mucosal morphology (**Figure 2A**).

Positive staining for occludin and ZO-1 was observed in the intestinal epithelium and glandular cells (**Figure 2C**). The percentage of cells staining positive for occludin differed significantly (P < 0.05 for all pairwise comparisons) between the control group (43.0 ± 4.1%), IGT group (15.6 ± 2.8%), and berberine group (23.4 ± 6.3%) (**Figure 2D**). Similarly, the percentage of cells staining positive for ZO-1 differed significantly (P < 0.05 for all pairwise comparisons) between the control group (38.8 ± 2.9%), IGT group (14.7 ± 2.9%), and berberine group (27.3 ± 4.5%) (**Figure 2D**). The IHC score was higher in the control group than in the IGT group (P < 0.05; **Supplementary Table 2**), and treatment with berberine was associated with improvements in IHC score (P < 0.05; **Supplementary Table 2**).

The positive staining for mucin was observed in intestinal mucus. **Figures 2C, E, F** showed that mucin expression was lower in the IGT group compared with the control group (P < 0.05) and that berberine increased mucin expression in IGT animals (P < 0.05).

### Effects of Berberine on Inflammatory Markers

**Figure 3:**
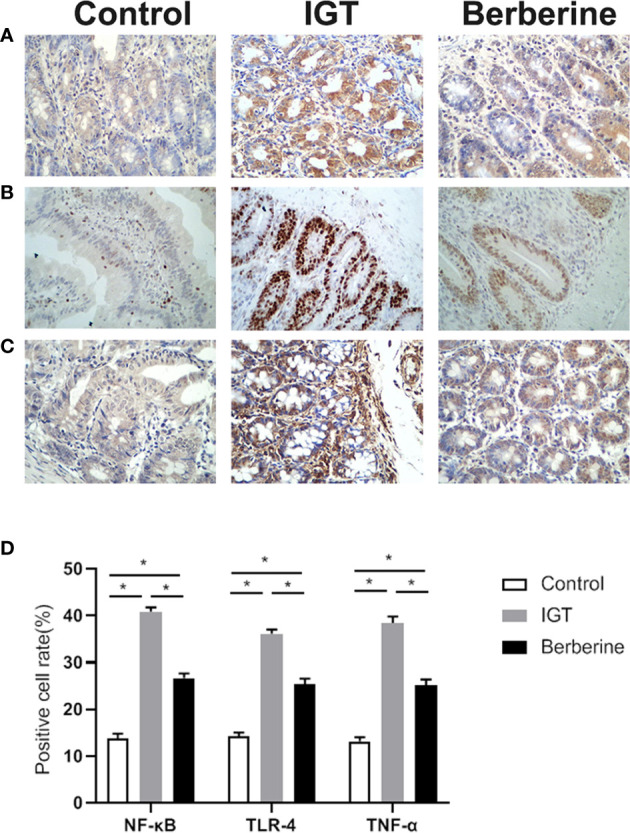
Representative immunohistochemistry images of **(A)** TNF-α, **(B)** NF-κB, and **(C)** TLR-4. **(D)** Statistical analysis of inflammatory factors expression by immunohistochemistry. *P < 0.05. Control group (n=5), IGT group (n=10), and berberine group (n=10).

Intestinal tissues were examined for the inflammatory markers TNF-α, NF-κB, and TLR-4 (**Figures 3A–D**). The IGT group displayed higher levels of TNF-α, NF-κB, and TLR-4 compared with the Control group. Berberine decreased inflammation compared with the IGT group, but without reaching the values of the control group.

### Effects of Berberine on GLP-1 and GLP-2 Levels

**Figure 4:**
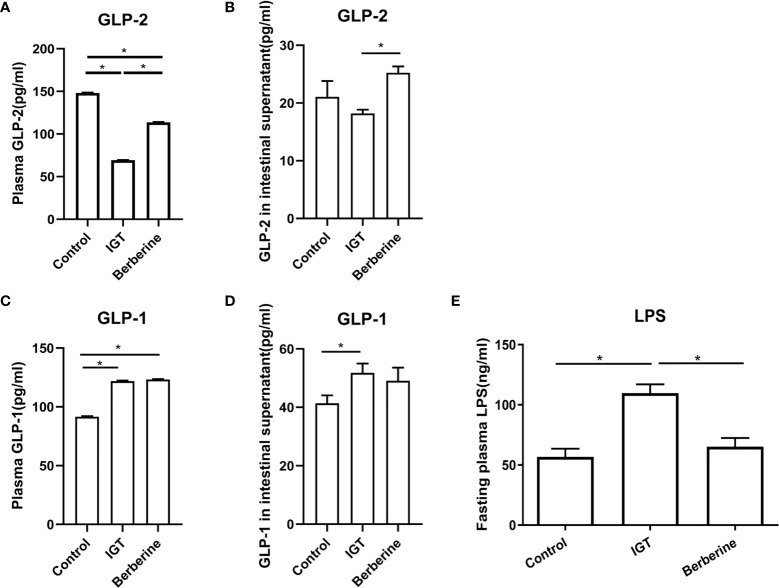
Effects of berberine on the levels of glucagon-like peptide-1 (GLP-1), glucagon-like peptide-2 (GLP-2), and lipopolysaccharide (LPS). **(A)** Fasting plasma GLP-2 levels. **(B)** Glutamine-induced GLP-2 secretion from ileal tissue. **(C)** Fasting plasma GLP-1 levels. **(D)** Glutamine-induced GLP-1 secretion from ileal tissue. **(E)** Fasting plasma LPS levels. *P < 0.05. Control group (n=5), IGT group (n=10), and berberine group (n=10).

Fasting plasma GLP-2 level was reduced in the IGT group in comparison to the control group (P < 0.05) but was partially restored by treatment with berberine (P < 0.05; **Figure 4A**). Glutamine-induced GLP-2 secretion from ileal tissue was not significantly different between the control and IGT groups but was enhanced after treatment with berberine (P < 0.05; **Figure 4B**). Fasting plasma GLP-1 level and glutamine-induced GLP-1 secretion from ileal tissue were both higher in the IGT group than in the control group (P < 0.05) but were not affected by treatment with berberine (**Figures 4C, D**).

### Effects of Berberine on Plasma LPS Levels

Fasting plasma LPS level after 3 weeks of the intervention was significantly higher in the IGT group than in the control group (P < 0.05; **Figure 4E**). However, treatment with berberine resulted in a reduction in LPS (P < 0.05) to a level comparable with that in the control group (**Figure 4E**).

### Effects of Berberine on the Structure of the Fecal Microbiota

**Figure 5:**
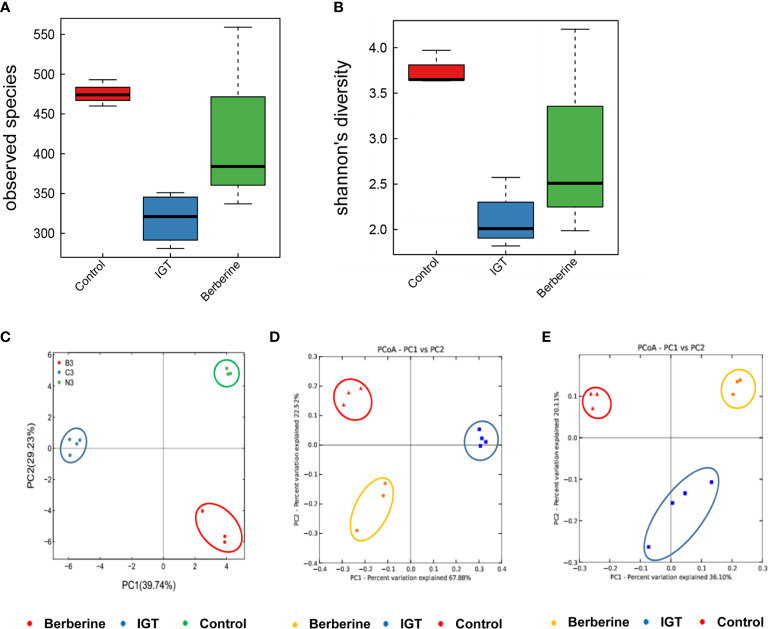
Effects of berberine on the structure of the fecal microbiota. **(A)** Observed species index. **(B)** Shannon diversity index. **(C)** Principal component analysis 3 weeks after the intervention. **(D)** Weighted UniFrac significance test 3 weeks after the intervention. **(E)** Unweighted UniFrac significance test 3 weeks after the intervention. Control group (n=3), IGT group (n=4), and berberine group (n=3).

High-quality pyrosequencing technology was used to determine the structure of the fecal microbiota. A total of 528,887 usable raw sequences (an average of 31,111 sequences per sample) were generated from 17 samples. The high-quality sequences were delineated into 1,664 OTUs at a similarity cutoff of 97%. Shannon and observed species diversity curves demonstrated that most of the microbial diversity in each sample was captured with the current sequencing depth (**Supplementary Figure 1**). Calculation of the Shannon diversity index and observed species index revealed that species richness (as indicated by the observed species index) and diversity (as shown by the Shannon diversity index) were lower in the IGT group than in the control group (**Figures 5A, B**). Furthermore, treatment with berberine partially restored the richness and diversity of the gut microbiota (**Figures 5A, B**).

PCoA was applied to visualize the overall structural changes in the gut microbiota. Weighted and unweighted UniFrac significance tests demonstrated that there were distinct structural differences between groups after 3 weeks of intervention (**Figures 5C–E**).

### Key Phylotypes of the Gut Microbiota Modulated by Berberine

**Figure 6:**
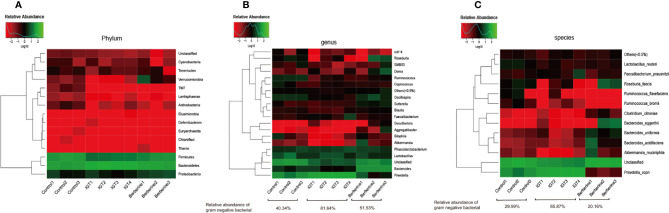
Gut microbial compositions in the different groups. **(A)** Phylum level. **(B)** Genus level. **(C)** Species level. Control group (n=3), IGT group (n=4), and berberine group (n=3).

The dominant phyla were *Bacteroidetes*, and *Firmicutes* followed by *Proteobacteria* and *Verrucomicrobia*. *Bacteroidetes* were significantly increased, and *Firmicutes* distinctly decreased in rats with IGT (in comparison to the control group), but berberine reversed these changes (**Figure 6A**). At the genus level, treatment with berberine was associated with enrichment of *Bacteroides*, *Oscillospira*, *Akkermansia*, *Aggregatibacter, Clostridium, Roseburia*, and *Eubacterium* as well as inhibition of *Prevotella*, which was the main component of the IGT group (**Figure 6B**). At the species level, the IGT group exhibited an increased abundance of *Prevotella copri*, a common Gram-negative bacterium in humans, and a decreased abundance of *Akkermansia muciniphila*, *Lactobacillus reuteri, and Bacteroides caccae* in comparison to controls (**Figure 6C**). Berberine improved the structure of the gut microbiota, as well as the abundance of Gram-negative bacteria (**Figure 6C**).

## Discussion

Prediabetes is a high-risk state for the development of T2DM (8). The risk of prediabetes progressing to T2DM can be reduced by lifestyle changes and drug-based interventions, including metformin, thiazolidinediones, and α-glucosidase inhibitors (8). Several lines of evidence suggest that incretin-based therapies, including GLP-1 receptor agonists and dipeptidyl peptidase-4 inhibitors, may stabilize or reverse the β-cell loss and slow or prevent the progression of prediabetes (35–37). In addition, the use of Chinese herbs in the prevention and treatment of diabetes has received increasing interest (38, 39).

Berberine, one of the main components of Huanglian Jiedu Decoction, is widely used in the treatment of colitis and other intestinal diseases (40). Berberine has been reported to have beneficial effects on energy metabolism, insulin resistance, and endotoxemia, both in diabetic rats and patients (41). Our previous study found that berberine decreased blood glucose level and insulin resistance in rats with T2DM induced by a high-fat diet (31). Previous studies also reported that berberine could improve the blood markers and pathophysiological changes of diabetes in patients and animal models (42–48). In the present study, the incidence of T2DM was significantly lower in the berberine group than in the IGT group, and 40% of rats in the berberine group reverted to having an NGT. Moreover, treatment with berberine led to significant improvements in blood glucose level, blood lipid levels, and insulin resistance, as supported by the literature (42–48). To our knowledge, this is the first study to demonstrate that berberine can prevent the progression of prediabetes to T2DM in rats. Since berberine is poorly absorbed from the intestine (49), we suggest that berberine may act directly on the gastrointestinal mucosa or gut microbiota. Therefore, we speculate that berberine prevents the progression of prediabetes to T2DM by improving intestinal permeability, GLP-2 secretion, and the structure of the gut microbiota and decreasing insulin resistance.

Risk factors associated with prediabetes include dyslipidemia, obesity, insulin resistance, and chronic low-grade inflammation. An increased level of plasma LPS, one of the cell wall components of Gram-negative bacteria, has been shown to be closely related to the chronic inflammation of diabetes (41). Compromised gut barrier function may be an important factor underlying the increase in LPS level, which aggravates inflammation and insulin resistance. Furthermore, LPS-induced inflammation may be related to abnormal GLP-2 function and changes in the gut microbiota (49). GLP-2 can protect the intestinal mucosal barrier by increasing the number of tight junctions between cells, promoting regeneration and growth, enhancing blood supply, and reducing damage (17, 22). Recent evidence suggests that GLP-2 is also closely related to glucose and lipid metabolism (50). Therefore, GLP-2 may function as a bridge between abnormal intestinal function and obesity-related diseases such as T2DM.

Previous studies have found that plasma GLP-2 levels in patients with T2DM are significantly lower than those in people without T2DM (22), but the cross-sectional design of these studies did not allow determination of whether the fall in GLP-2 level occurs before the development of T2DM or is secondary to it. In the present study, we measured the GLP-2 level in ZDF rats at different stages of glucose tolerance and found that the fasting plasma GLP-2 level gradually decreased from NGT to IGT to T2DM. It has been reported that the decline in GLP-2 level in ZDF rats with IGT or T2DM may be due to an impairment in the number and/or function of intestinal L cells (51). Our study found that treatment with berberine increased the secretion of GLP-2. In agreement with our findings, it has been reported that berberine enhances the expression of the proglucagon gene (the precursor gene for GLP-1 and GLP-2) and the proliferation of intestinal L cells in normal and diabetic rats (52). Therefore, berberine may increase GLP-2 levels by modulating intestinal L cell proliferation and proglucagon gene expression. We also found that fasting plasma levels of GLP-1 increased gradually from NGT to IGT to DM. The association of T2DM with enhanced glucose-induced or postprandial GLP-1 secretion has also been reported in other studies (52–54). The increased secretion of GLP-1 may reflect an increased sensitivity of intestinal L cells to nutrients. However, we did not find significant effects of berberine on GLP-1 secretion both in the fasting state and in the glutamine-induced state. It remains unclear why GLP-1 and GLP-2 levels in ZDF rats changed differently during the progression of diabetes. This is an interesting result because the traditional view is that GLP-1 and GLP-2 are released simultaneously from intestinal L cells at a ratio of 1:1. There may be some compensatory mechanisms that induce GLP-1 secretion to counteract high glucose levels at the expense of GLP-2 secretion.

Occludin and ZO-1 are components of tight junctions and mucin forms the skeleton of the intestinal mucus to cover and protect the intestinal tract from self-digestion or microorganisms. They are often used to evaluate intestinal mucosal barrier function (55). In our study, rats with T2DM exhibited damage to the intestinal mucosal barrier, as shown by the down-regulated expression of mucin, occludin and ZO-1. This finding is consistent with the observed increase in LPS level, which would reflect the translocation of bacteria and their components from the intestine to the blood circulation, as well as chronic inflammatory response as signified by up-regulated expression of TLR-4, NF-κB, and TNF-α. Activation of the GLP-2 receptor in intestinal epithelial cells is thought to reduce the translocation of bacteria and bacterial components into the blood by regulating mesenteric blood flow, promoting epithelial cell regeneration, reducing apoptosis, decreasing intestinal leakage, and enhancing intestinal mucosal barrier function (17). We observed that the progression of T2DM in ZDF rats was associated with an increase in the plasma LPS level and a decrease in the GLP-2 level. Furthermore, treatment with berberine resulted in both an increase in GLP-2 secretion and a decrease in the LPS level.

We also found that the general trend in the LPS level during the progression of T2DM was consistent with the change in the relative abundance of Gram-negative bacteria in the feces. Since LPS is a cell wall component of Gram-negative bacteria, an improvement in the structure of the gut microbiota would be expected to lead to a decrease in LPS production. Our findings indicate that berberine markedly improved the structure of the gut microbiota after 3 weeks. The increased abundance of *Prevotella*, especially *Prevotella copri*, may contribute to high-fat diet-induced obesity. *Prevotella copri* is a Gram-negative bacterium that may directly affect host metabolism. For example, in mice fed a high-fat diet, *Prevotella copri* was found to induce insulin resistance, aggravate glucose intolerance, and augment circulating levels of branched-chain amino acids (56). We also observed consistency between the change in insulin resistance and a relative abundance of *Prevotella copri* in ZDF rats. Notably, the improvement in insulin resistance and glucose intolerance following treatment with berberine was accompanied by a decrease in the abundance of *Prevotella copri*. Furthermore, short-chain fatty acid producers such as *Bacteroides*, *Clostridium*, *Roseburia*, and *Akkermansia* were also affected by berberine. Short-chain fatty acids may promote a healthy weight by reducing appetite and/or altering energy metabolism. *Akkermansia*, particularly *Akkermansia muciniphila*, has been shown to be a favorable factor for reducing body weight, improving blood glucose level, and relieving insulin resistance. In addition, the bacteria may mediate, at least in part, the therapeutic effects of metformin and bariatric surgery on T2DM (57). The improvement in the composition of the gut microbiota by berberine may be responsible, at least in part, for slowing the progression of prediabetes to T2DM. In addition, *Firmicutes* have been reported to be increased in obesity (58), but a recent study casts doubts about a firm association of the *Firmicutes/Bacteroides* ratio with specific health statuses, including obesity (59). Additional studies are necessary to examine this.

In this study, a decrease in food intake and a maintenance of body weight were observed with berberine. The maintenance of body weight despite reduced food intake could be due to a better intestinal health favoring a more efficient uptake of the nutrients, combined with changes in the gut microbiota. This hypothesis will have to be examined specifically in future studies.

In this study, berberine was administered at a dose of 100 mg/kg/d for 3 weeks, based on previous studies in rats (28, 31, 32, 47). The antidiabetic effect of berberine is dose-dependent. Some studies have showed that the berberine at a low dose of 50mg or 60mg/kg had no significant antidiabetic effect (46, 52). Other studies have reported that berberine at a higher dose of 120mg to 300mg had beneficial effects on glucose metabolism with few side effects (25, 29, 46, 48, 52, 53).Future studies should examine the effect of various doses of berberine on GLP levels in relation to T2DM. Additionally, according to available equations, the dose of 100 mg/kg/day would correspond to a dose of 13.8 mg/kg/day in humans (60 kg) (60). It was previously demonstrated that the dose of berberine that can be administrated in patients with type 2 diabetes is 900mg-1500mg/day, which were effective and safe without severe gastrointestinal adverse effects (61, 62). Berberine possesses a low oral bioavailability, however, it has exhibited the metabolites of berberine showed similar bioactivities and the concentrations of its major metabolites were at relatively high levels (63). Furthermore, in regard to the treatment duration, previous studies have shown that it would take two weeks to get the significant effect of improving insulin resistance (28, 31, 47), and other previous studies showed more treatment duration of five to eight weeks (25, 29, 32, 46, 48, 52, 53). In present study, it has been shown that 3 weeks was sufficient to reach a plateau in the effects of berberine, at least in rats, but it would have to be verified in humans. Difference in dosages and treatment durations, and low rates of absorption after oral berberine administration might explain this discrepancy.

The pathophysiological changes in the liver and kidney were not examined. Previous studies reported improvements in fatty liver (42–44, 48) and diabetic nephropathy (45–47) with berberine treatment. It is true that berberine can be toxic to the liver and kidneys (64), but the present study used a berberine dosage known to be therapeutic in rats (25, 28, 29, 31, 32, 48, 52, 53). Nevertheless, the liver and kidney changes will be examined in future studies. Future studies will also examine the pathophysiological changes in the intestine, complete panels of inflammatory and immune markers, and short-chain fatty acids production and levels. Finally, this study was observational and was not designed to determine the exact mechanisms involved in the effects of berberine. Future studies will be designed to do so.

To summarize, our study is the first to show that the GLP-2 level decreases before the occurrence of T2DM in ZDF rats. Furthermore, treatment with berberine slows the progression of prediabetes to T2DM, and this effect may be related to improvements in intestinal GLP-2 secretion, the intestinal barrier, and the structure of the gut microbiota.

## Data Availability Statement

The original contributions presented in the study are publicly available. This data can be found here: NCBI PRJNA681546.

## Ethics Statement

The animal study was reviewed and approved by Animal Ethics Committee of Tianjin Medical University.

## Author Contributions

YW contributed to conception, analysis, and drafted the manuscript. HL contributed to the design, analysis of data and drafted the manuscript. MZ and YY participated in the conception, interpretation of data, and critically revised manuscript. HR, YK, and SW contributed to the design, acquisition of data and drafted the manuscript. JW contributed to the conception, analysis, and critically revised manuscript. YJ contributed to the design, acquisition of data and drafted the manuscript. JY contributed to design, interpretation of data, and critically revised manuscript. CS contributed to the conception, interpretation of data, and critically revised manuscript. All authors contributed to the article and approved the submitted version.

## Funding

This work was supported by the National Natural Science Foundation of China (81700631), the Science and Technology Program of Tianjin (17ZXHLSY00040), the Science and Technology Development Fund of Tianjin Education Commission for Higher Education (2016YD05).

## Conflict of Interest

The authors declare that the research was conducted in the absence of any commercial or financial relationships that could be construed as a potential conflict of interest.
