# Supplementary material for: Berberine Slows the Progression of Prediabetes to Diabetes in Zucker Diabetic Fatty Rats by Enhancing Intestinal Secretion of Glucagon-Like Peptide-2 and Improving the Gut Microbiota
Source: Front Endocrinol (Lausanne). 2021 May 7;12:609134. doi: 10.3389/fendo.2021.609134 (PMC8138858; doi:10.3389/fendo.2021.609134)
Supplement: Supplementary file 1 [file DataSheet_1.docx]

Supplementary Material

# Supplementary Figures and Tables

## Supplementary Figures


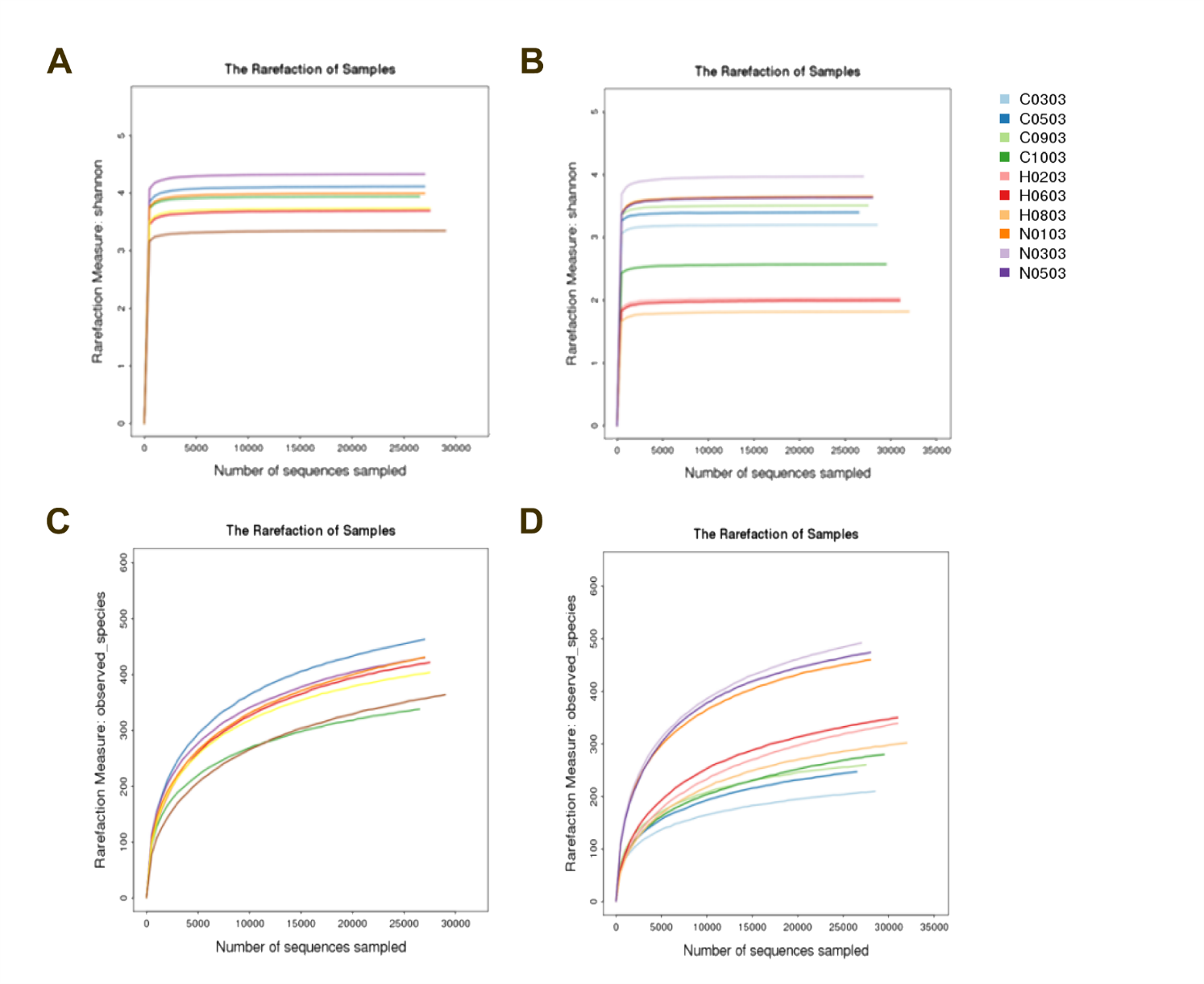


**Supplementary Figure 1. Shannon curves and observed species curves.** **(A)** Shannon curves at the start of the study (when all rats had normal glucose tolerance). **(B)** Shannon curves 3 weeks after the intervention. **(C)** Observed species curves at the start of the study (when all rats had normal glucose tolerance). **(D)** Observed species curves 3 weeks after the intervention.

A

B

C

**Supplementary Figure 2. Changes in metabolic markers over time.** **(A)** Changes in food intake over time. **(B)** Changes in body weight over time **(C)** Changes in blood glucose levels over time. All data are shown as means ± standard deviations. Control: normal control; IGT: impaired glucose tolerance; Berberine: impaired glucose tolerance treated with berberine; *P<0.05, **P<0.01 vs. IGT; ^#^P<0.05, ^##^P<0.01 vs. Control.

## Supplementary Tables

**Supplementary Table 1. Plasma lipid, hepatic and renal functions after treatment by berberine**

|  | ALT (IU/L) | AST (IU/L) | BUN (mmol/l) | CR (umol/l) | UA (umol/l) | CHO (mmol/l) | TG (mmol/l) | HDL (mmol/l) |
| --- | --- | --- | --- | --- | --- | --- | --- | --- |
| Control | 57.99±1.17 | 161.28±1.29 | 7.90±0.45 | 34.3±3.11 | 60.06±16.10 | 2.23±0.69 | 1.52±0.02 | 0.4±0.01 |
| IGT | 86.12±1.19^*^ | 140.99±1.39^*^ | 11.63±3.07^*^ | 22.83±2.74^*^ | 76.69±23.51 | 3.79±0.47^*^ | 4.48±1.37^*^ | 1.82±0.25^*^ |
| Berberine | 70.42±1.20^*#^ | 102.75±1.16^*#^ | 9.64±1.38^*^ | 23.6±2.20^*^ | 50.57±12.75^#^ | 3.12±0.52^*#^ | 2.64±0.7^*#^ | 1.33±0.33^*#^ |
| P value | 0.001 | 0.010 | 0.012 | 0.000 | 0.012 | 0.000 | 0.000 | 0.000 |
| F value | 10.336 | 5.593 | 5.337 | 37.201 | 5.334 | 19.330 | 17.793 | 62.485 |

^*^:P<0.05 vs. control group, ^#^:P<0.05 vs. IGT group. ALT: Alanine aminotransferase; AST: Aspartate aminotransferase; BUN: Blood urea nitrogen; CR: Serum creatinine; UA: Blood uric acid; CHO: Cholesterol; TG: Triglyceride; HDL: High density lipoprotein.

**Supplementary Table 2. IHC Score of ZO-1 and occluding**

|  | ZO-1 | occludin |
| --- | --- | --- |
| Control | 6 (6, 6) | 6 (6, 6) |
| IGT | 2 (2, 2)^*^ | 2 (2, 2)^*^ |
| Berberine | 4 (4, 4.5)^#^ | 4 (3.5, 4.5)^#^ |

^*^: P<0.05 vs control group, ^#^:P<0.05 vs IGT group.
